# Supplementary material for: Electrically switchable and tunable infrared light modulator based on functional graphene metasurface
Source: Nanophotonics. 2023 Mar 22;12(9):1797–807. doi: 10.1515/nanoph-2023-0048 (PMC11501403; doi:10.1515/nanoph-2023-0048)
Supplement: Supplementary file 1 — Supplementary Material Details [file j_nanoph-2023-0048_suppl_001.docx]

Electrically Switchable and Tunable Infrared Light Modulator based on Functional Graphene Metasurface

Wei Luo^1^, Syeda Aimen Abbasi^1^, Shaodi Zhu^1^, Xuejin Li^2^, Ho-Pui Ho^1^, Wu Yuan^1^^*^

^1^Department of Biomedical Engineering, The Chinese University of Hong Kong, Hong Kong, 999077, China

^2^School of Science and Engineering, The Chinese University of Hong Kong, Shenzhen, Guangdong, 518172, China

**Keywords:** Optical modulator; Electrical switching and tuning; Polarization converter; Graphene; Functional metasurface.

**Abstract:**

Graphene is emerging as an ideal material for new-generation optoelectronic devices. In this paper, a novel graphene metasurface-based electrically switchable and tunable infrared light modulator has been proposed and theoretically studied. The functional modulator comprises a monolayer graphene sheet sandwiched in a Fabry-Perot (FP) like nanostructure consisting of a metal reflector, a dielectric spacer, and an ellipse patterned anisotropy antenna layer. As a result of the photon localization effect of the guided-mode resonance (GMR) in the FP structure, the graphene electroabsorption can be significantly enhanced to enable a high-performance light modulator. By fine-tuning the Fermi energy (*E_f_*) of graphene via controlling its bias-gate voltage, the proposed modulator can switch between a perfect absorber and a reflective polarization converter of high conversion efficiency (i.e., >90%) at 1550 *nm*. The conversion mechanism and the geometric dependences of the infrared light modulator have been investigated. We further demonstrated the tunability of the highly-efficient polarization converter over a broad spectrum by adjusting the real dispersion of *E_f_*. Our design concept provides an effective strategy for customizing novel optoelectronic devices by combining an electrically-tunable 2D material with a functional metasurface.

^*^Corresponding author

Tel: 852- 3943 0495 E-mail: [wyuan@cuhk.edu.hk](mailto:wyuan@cuhk.edu.hk) (W. Yuan)

1. **Introduction**

Polarization is an essential property of electromagnetic (EM)/light waves. The effective manipulation of the polarization state of light is essential and highly desired in many applications, including sensing, imaging, and communication [1-4]. Traditionally, polarization manipulation can be achieved by using optical gratings and total internal reflection in crystals or employing the Faraday effect, birefringence materials, etc [5-7]. However, conventional methods usually require a long propagation distance to accumulate enough phase shift, leading to a convector of large form sizes, high loss, and low efficiency, which significantly limits its application. Metasurface, as a two-dimensional artificial material, can utilize a plasmonic resonance to generate abrupt change in phase and amplitude of light, thus the polarization, and attract significant interest, being regarded as a promising modulator. So far, considerable investigations have been taken to develop various polarization converters using metallic metasurface [8, 9]. Nevertheless, due to the restricted flexibility of permittivity of the components, most of the modulators operate well at the specifically designed wavelength range [10-12]. Therefore, it is highly desired to construct a tunable structure to realize an active modulation of the converter in an expected region.

Fortunately, graphene satisfies the requirement due to its exotic property [13-15]. As the first truly two-dimensional (2D) material to be found and isolated successfully from graphite, graphene is an atom-level-thick sheet layer whose carbon atoms are arranged in a planar honeycomb lattice. Thus, charge transport in graphene can be characterized as a 2D electronic system [16]. It has been demonstrated that, compared to silicon and III-V semiconductors, the specific band structure of graphene results in various superior properties, such as ultra-high thermal conductivity, high optical damage threshold, and strong optical nonlinearities. In the mid-infrared and terahertz (THz) ranges, graphene acts like a metal supporting highly confined surface plasmons, leading to enhanced optical absorption and strong light-graphene interaction [17-19]. Therefore, considerable efforts have been devoted to develop various dynamic meta-devices with different functions, such as spectrum manipulation, wavefront shaping, polarization control, and frequency conversion in near/far fields and global/local ways [20-22]. Specifically, at the near-infrared wavelength region where *ℏω_o_>2E_f_* (*E_f_* denotes the Fermi level of graphene), graphene behaves like an absorptive dielectric, while for *ℏω_o_<2E_f_*, it functions as a lossless material [23, 24]. More importantly, graphene owns the original gate-voltage-dependent optical feature, that the chemical potential can be controlled by turning the external bias voltages, which enables one to construct an ultracompact and active tunable optical modulator [18, 25]. However, due to the fine structure constant α=*e^2^/ℏc*, the absorption efficiency of monolayer graphene is only about 2.3% with a single-pass illumination, which significantly restricts its relevant application [26, 27].

Therefore, the absorption enhancement of graphene is critical to realize efficient plasmonic modulation with high tunable depth [28, 29]. In the mid to far-infrared frequency range, doped graphene acts like a metal and sustains strong surface plasmon resonances, which is utilized to improve electroabsorption [30-33]. Ye *et al.* demonstrated multiple approaches to enhance multi-band terahertz absorption based on composite graphene and metal microstructures [34, 35]. Meanwhile, plasmonic resonance cannot be supported for the visible and near-infrared regions due to its limited doping level, and the graphene performs like an absorptive dielectric. Thus, the absorption enhancement is generally achieved by introducing various photonic resonant structures, such as localized plasmon resonance generated by attenuated total reflection (ATR) or Fano resonance, Fabry-Perot (FP) cavity, and critical coupling of guided-mode to couple graphene with dielectric or metallic resonance structure, etc [36-39]. Cui *et al.* [40, 41] theoretically proposed a graphene-based absorber at the near-infrared frequency by hiring a hybrid Tamm plasmonic system, and both the guided-mode resonance (GMR) and Tamm plasmon polaritons can be generated efficiently. With the implementation of a magnetic dipole resonance, Chen *et al.* [42] numerically demonstrated a broadband near-infrared absorption enhancement of graphene and its electrically switchable effect. In 2022, Nong *et al.* studied the active modulation of graphene electroabsorption [43]. According to their work, borophene plasmons induce the localized electrical field. Then the center wavelength of graphene absorption can be dynamically controlled by gate-tuning the resonant wavelength of borophene plasmons. However, the mentioned works mainly focus on the enhancement and electrically tunable modulation of the absorption at different wavelength ranges. Combining these unique merits with a functional metasurface can extend graphene’s application from the conventional absorption modulator into the controllable electrical modulator.

Herein, we propose an electrically switchable and tunable near-infrared light modulator centered at a communication frequency of 1550 nm. It is realized by introducing a monolayer graphene sheet with a functional metasurface. The FP-like metasurface supports dielectric mode resonances. Then the electromagnetic field is strongly confined within the structure, and the electroabsorption of graphene can be dramatically enhanced. Then by artificially switching graphene between a lossy and lossless material or electrically tuning its optical dielectric, the modulator can work as an absorber or a reflector. Further, the functional antenna design can achieve a factitiously controllable polar converter. Moreover, the modulator can be processed in real-time and be convenient for engineering.

1. **Electrically switching the modulator between an absorber and a polarization converter**

*2.1 Structure design and working status of the absorber*

The schematic diagram of the modulator proposed in this work is shown in Fig. 1. The structure cell comprises a full monolayer graphene sheet sandwiched between a spacer layer and an antenna block patterned with an elliptical nanohole. The symmetry axis of the ellipse pattern is rotated 45^o^ counterclockwise from the x-axis direction, indicating an anisotropy in the x-y plane. Here, *a* and *b* represent the semi-length of the minor and major axis of the elliptical nanohole pattern. *h_1_* and *h_2_* denote the thicknesses of the antenna nanohole and spacer layer. The bottom silver layer serves as a reflector with a thickness of 200nm, bigger enough than the skin depth to suppress the wave transmission.

The insert shows the electromagnetic wave’s coordinates. The light is a normal incidence along the z-axis, vertically irradiating from the top, and the nanostructure supports several GMRs. When these two wavevectors match, the incident wave will couple with the inherent guided mode, meeting the resonance condition. As a result of photon localization from the resonance, the absorption of the graphene would be significantly enhanced. Thus, the reflection is generated with graphene’s electrically switchable and tunable optical dielectric. Furthermore, a converter can be realized with the implementation of modulation by the anisotropy antenna layer. Since the absorption of graphene is mainly due to the bias-gate-voltage dependent direct interband transition, a gate voltage is added to realize the dynamic switching between different statuses, i.e., absorber and polarization converter.


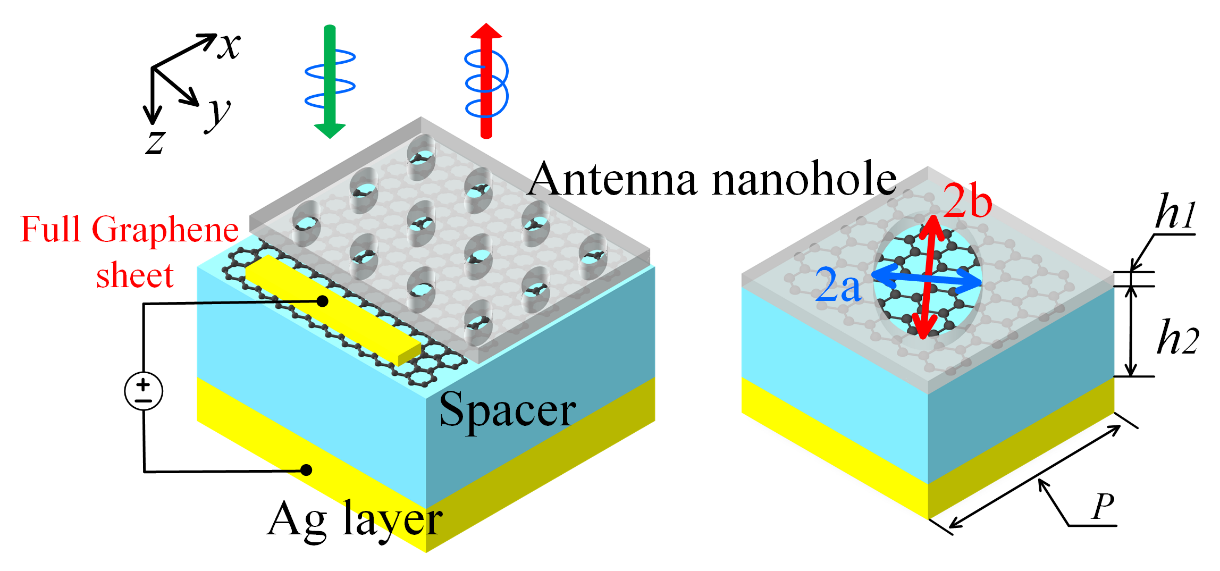


Fig1. Schematic of the infrared light modulator consisting of a full monolayer graphene sheet with electrically tunable chemical potential and a FP-like ellipse-patterned metasurface. *a*=200 *nm*, *b*=600 *nm*. The bias gate voltage and the polarization status of the incident and reflected light are also shown.

The finite-element method is applied to investigate the property of the proposed metasurface using the COMSOL software. In the theoretical simulation, graphene is treated as an effective surface conductor for its ultra-thin thickness, and the Kubo formalisms can describe the surface conductivity of graphene (σ_gra_) with the summary of the intra-band term (σ_intra_) and the inter-band term (σ_inter_) as the following [41, 44]:

. (1)

Here, *e*, *ω*, *k_B_*, *T*, ℏ, and *E_f_* represent the electron charge, the frequency of incident light, the Boltzmann constant, the temperature in kelvin, the reduced Planck constant, and Fermi energy (or chemical potential), respectively. *τ* denotes the momentum relaxation time for the charge carrier scattering. It relates with the carrier mobility *μ* as *τ=μE_f_/(ev_f_ ^2^)*, where *v_f_* is the Fermi velocity of about 10^6^ m/s [45]. Previous reports showed that the carrier mobility of graphene on SiO_2_ substrate could reach 4×10^4^ cm^2^/(V.s) at room temperature. Note that the carrier concentration will not increase infinitely with the increase of *τ*. It has a critical saturation value when obtaining the highest absorption efficiency. Therefore, either at the low or high frequency resonance, the relaxation time variation causes little wave shift, which indicates that the dynamical tunability is only related to the Fermi level of graphene. Hence, the *τ* is set with the value of 0.5 *ps* in the simulation to ensure the credibility of results [41, 46, 47]. As for an electromagnetic wave propagating inside the graphene layer with a finite thickness Δ, it has a relation of *Δ×H=-iω**ℇ_0_E+J=-iωℇ_0_ℇ_g_E*. Here *J=E_||_σ/Δ*. *E_||_* is the electric field along the graphene surface. Therefore, the anisotropic relative permittivity tensor of graphene can be described as:

$\varepsilon_{g}=\left(\begin{matrix} 1+i\sigma/\left(\omega\varepsilon_{0}t_{g}\right) & 0 & 0 \\ 0 & 1+i\sigma/\left(\omega\varepsilon_{0}t_{g}\right) & 0 \\ 0 & 0 & 1 \end{matrix}\right)$ (2)

where *ℇ_0_* is the graphene permittivity, and *t_g_* stands for the thickness of the monolayer graphene sheet (typically 0.34 *nm*). A silver layer is used as the reflector, and its dielectrics permittivity is described by the Drude model ℇm(ω)=ℇ∞-(ω_p_)^2^/(ω^2^+jωγ). ℇ∞ is the dielectric constant at the infinite frequency, and the corresponding electron collision and bulk plasma frequencies are 0.018 *eV* and 9.1 *eV*, respectively. The proportional ratio of the radiation absorbed by the monolayer graphene is dependent on the electrical energy incident upon its surface and can be obtained as [40]:

$abs=\frac{\iiint_{V} \omega(x,z)dV}{0.5c\varepsilon_{0}\left|E_{inc}\right|^{2}S\cos\theta}$ (3)

Here *ω(x, z)* is the power dissipation density that depends on a combination of electric field and material loss. In the simulation, periodical boundary conditions are applied to a single calculation cell to approximate an infinite array. The antenna layer and spacer thicknesses are designed to be 280 *nm* and 520 *nm* with dielectric constants of 1.48 and 1.45. The background is assumed to be air. A periodicity (*P*) of 1258 *nm* is used to suppress the high-order diffraction at the wavelength of 1550 *nm*. *E_f_* is set as 0.3 *eV* at the beginning, where the graphene exhibits as a loss material. Only x-polarized incident light is considered in this study to prove the design concept.


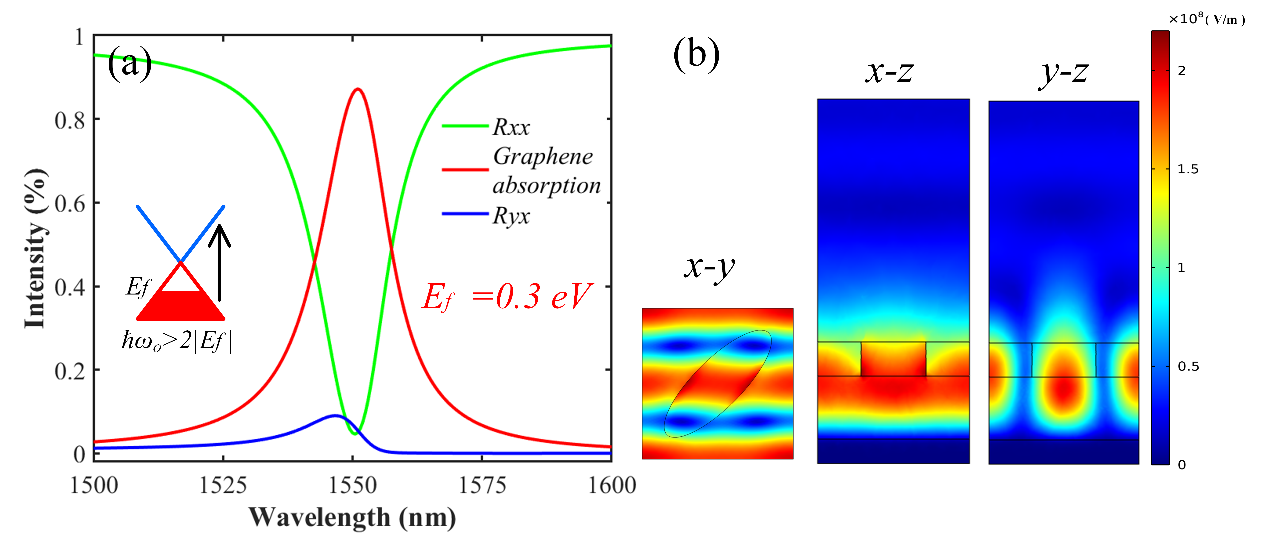


Fig. 2. (a) Simulation intensity of *R_xx_*, *R_yx_,* and absorption of the monolayer graphene under normal *x*-polarized incidence (τ=0.5 *ps*). The insert indicates direct interband transition. (b) Normalized electric field distributions of the structure with an *x-*polarized incidence of a plane wave. Note that the subscripts *i* and *j* in coefficients *R_ij_* refer to the reflection and illumination polarization states.

Reflection coefficients of the co- (*R_xx_*) and cross- (*R_yx_*) polarization together with graphene absorption are shown in Fig. 2(a). The graphene absorption dramatically increases with declined *R_xx_*, indicating the reduction attributed to the enhanced graphene absorption. In contrast, the cross reflectivity (blue line) is suppressed. The detailed electric field distribution at the horizontal surface of the graphene sheet and the vertical planes (*x-z* and *y-z* planes) are shown in 2(b). The incident light forms a well-confined resonant mode, and there is no interference between the incidence and reflection, forming a typical standing wave profile. It also indicates that the modulation structure meets the critical coupling condition, where the leakage rate of the guided mode resonance out of the structure is equal to the absorption rate of that resonance in the absorptive materials. Moreover, the confined electric field localizes compactly at the spacer-antenna boundary where the graphene is deposited, leading to a greatly enhanced graphene-light interaction and further modulated status of the reflected light.

*2.2 Dependence of graphene dielectric on bias-gate controlled Fermi energy*

The enhanced graphene absorption mainly attributes to the strengthened induced electric current on its surface caused by the interband transition of electrons. It can be readily understood that, for the frequency region where *ℏω_o_>2E_f_*, the absorbed light is transferred into kinetic energy for electrons to realize the direct interband transition. While for *ℏω_o_<2E_f_*, the transition is suspended due to the declined optical energy (*ℏω_o_*), and then the graphene performs as a lossless material. Therefore, graphene’s Fermi energy (*E_f_*) can be adjusted by controlling the bias-gate voltage. It offers a practical approach to realizing the switchability and turnability of our modulator [48, 49]. Since the interband transition is reflected in the spectral properties of its dielectric, the real and imaginary parts of graphene dielectric permittivity under different Fermi energy are shown in Fig.3.


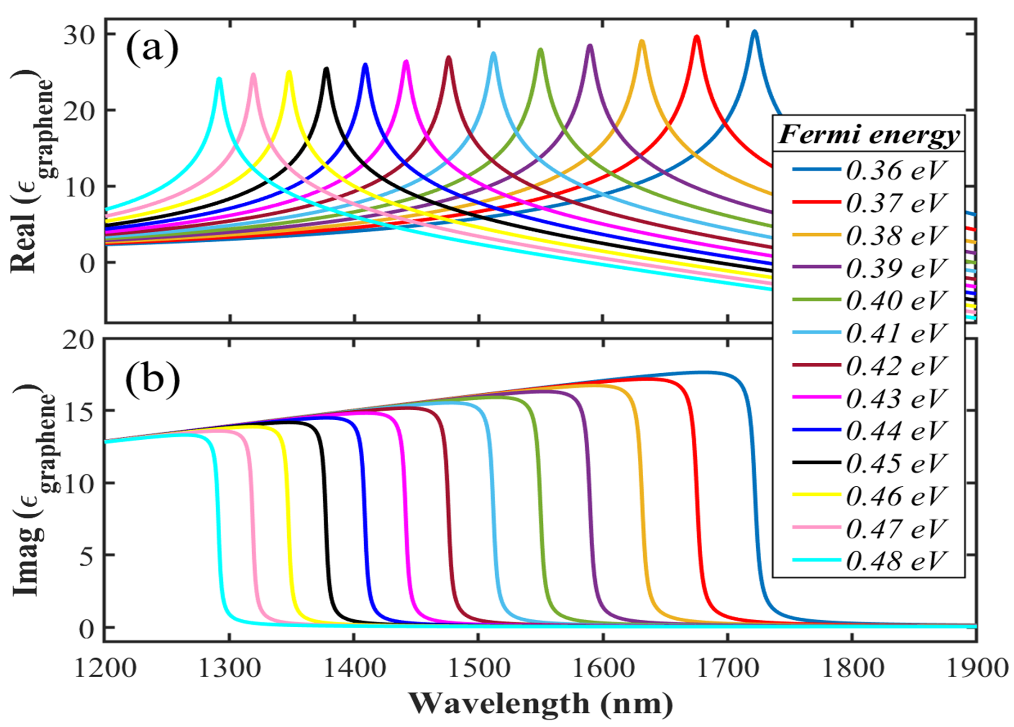


Fig. 3. The real (a) and imaginary (b) parts of dielectric coefficient of graphene under different Fermi energy.

The dielectric dispersion of graphene is quite different from conventional 2D materials due to its conduction and valence band meeting at the Dirac point where the Fermi level locates. Taking the *E_f_*=0.37 *eV* (solid red lines) as a typical analysis, the real and imaginary appear entirely different at two wavelength regions divided by an apparent boundary wavelength of about 1.68 *μm*. With the wavelength increasing across the boundary, the real part shows a sudden rise and rapid drop. At the same time, the imaginary performs a dramatic decline from a certain big value to almost zero, leading to a sharp peak in the real part and a sudden fall in the imaginary. Since the imaginary component indicates the absorption property of a material, graphene acts as a loss or lossless dielectric on the left or right side of the boundary. It also indicates that the wave region smaller or higher than 1.68 *μm* corresponds to the wavelength domain of *ℏω_o_>2E_f_* or *ℏω_o_<2E_f_*, individually. Thus, the boundary wave has a corresponding energy, i.e., interband transition energy of 0.74 *eV*. Further, with varying Fermi energy from 0.36 *eV* to 0.48 *eV*, the diving boundary shifts from 1.72 *μm* to 1.29 *μm*. As such, the lossy and lossless wavelength domains change reversely. Therefore, by adjusting the Fermi energy using the gate-bias voltage, the graphene can be switched between lossy and lossless material at a specific wavelength.

*2.3* *Working status of the reflector as polarization converter*

In the analysis of Fig.2, *E_f_* is set as 0.3 *eV* with corresponding interband transition located at 2070 *nm*, and the graphene layer behaves as an absorption dielectric. In contrast, when setting *E_f_* as 0.45 *eV*, the interband transition wavelength moves to 1380 *nm*, and graphene performs as a lossless material in the calculation range of 1500-1600 *nm*. A dramatically increased *R_yx_* at 1550 nm is found in Fig. 4(a) with a suppressed graphene absorption, indicating an efficiently converted cross-polarization component in the reflection. Mathematically, the polarization conversion ratio (PCR) is defined as:

$PCR=\left|Ryx\right|^{2}/\left[\left|Rxx\right|^{2}+\left|Ryx\right|^{2}\right]$, (4)

which is used to describe the conversion efficiency. The phase difference (PD) between *R_yx_* and *R_xx_* is deemed as *Δφ=φ_yx_-φ_xx_* implying all the possible polarization states (linear, circular, elliptic) of reflected light. When *cos(Δφ*)=±1, the polarization direction would be transformed to *y-*axis. If the magnitudes and phase shift satisfy the relationship *R_yx_=R_xx_* and *Δφ=2nπ**±π/2* (*n* is an integer), the reflected light turns to be a left- or right-handed circularly polarized. In other cases, it turns out to be elliptic polarization.


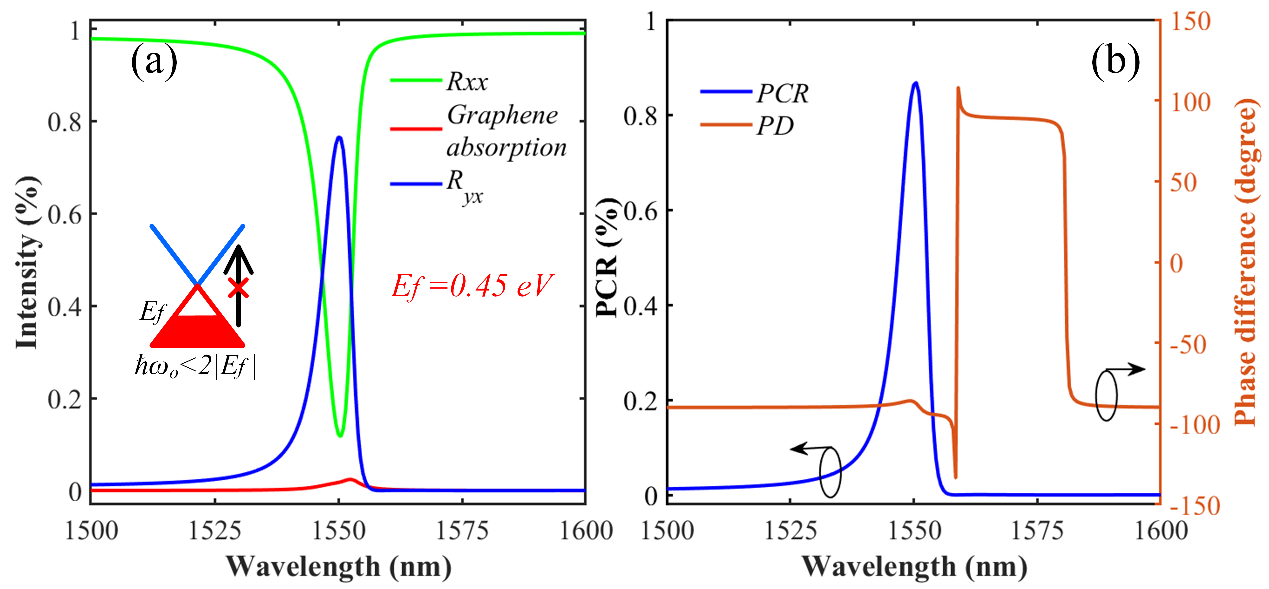


Fig. 4. (a) Co- and cross-polarization reflection intensity coefficients (*R_xx_* and *R_yx_*) of the proposed light modulator. The insert implies the suspended interband transition. (b) The PCR and PD curves.

The PCR and PD spectra are depicted in Fig. 4(b). The PD can only be either -90 or 90 (degrees), indicating the elliptic status of reflection. For the wavelength range with PCR=0, the reflection is a linear polarization with a phase change of *±π/2*, while for 1550 nm, the modulator turns out to be an elliptic polarization converter. Thus, the proposed graphene metasurface can be manually switched from the operational status of a perfect absorber to a polarization converter by adjusting the Fermi energy.

*2.4 Mechanism of the metasurface for polarization conversion*

To clarify the conversion mechanism, two mutually perpendicular symmetry axis, along the short and long-axis ellipse patterns, are defined as the *u*- and *v*-axis shown in Fig. 5(a). Then, the eigenmode response of the modulator, whose polarization is along the *u*- and *v*-axis, is studied. Since the incident EM (*e^ikzz^*) is decomposed into two orthogonal components, i.e., *E_u_^i^* and *E_v_^i^* in *u-v* coordinates expressed in equation (5), the reflection can be calculated as equation (6).

$E^{i}=\frac{1}{\sqrt{2}}\left(\hat{u}E_{u}^{i}+\hat{v}E_{v}^{i}\right)e^{ik_{z}z}$ (5) (6)

Here, the *û* and $\hat{v}$ denote the unit vectors along the *u-v* coordinates. Equations reveal that both the amplitude and phase are modulated. Then, with the eigenmode incident, the detailed reflectivity and PD are calculated and shown in Fig. 5. It is critical to find that, for each eigenmode, the polarization status is sustained while giving different responses in both amplitude and phase. For the *u-*polarization wave, it has a relatively small magnitude reflection but a faster phase transition. Moreover, the PD (*Δφ*) reaches a peak value at 1550 nm, shown in Fig. 5(b) as the green line, which indicates the dramatical phase accumulation at the wavelength point [33]. Therefore, the electric field of *x*-polarized incidence can be decomposed into *u* and *v* components to analyze the eigenmode response to each element. Then the reflection is the re-coupling of these two modulated components.


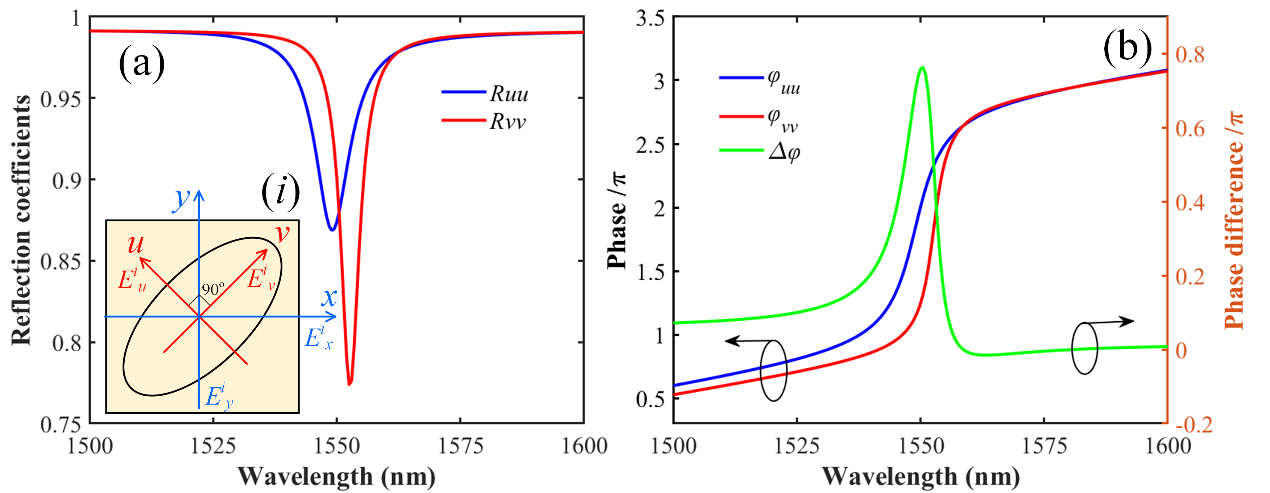


Fig. 5. (a) Reflection coefficients of the *u-* and *v-*polarization illustration, and (b) its corresponding phase response (left axis) and differences (right axis). The first and second subscripts in *R_ab_* represent the polarization status of the reflection and incident.


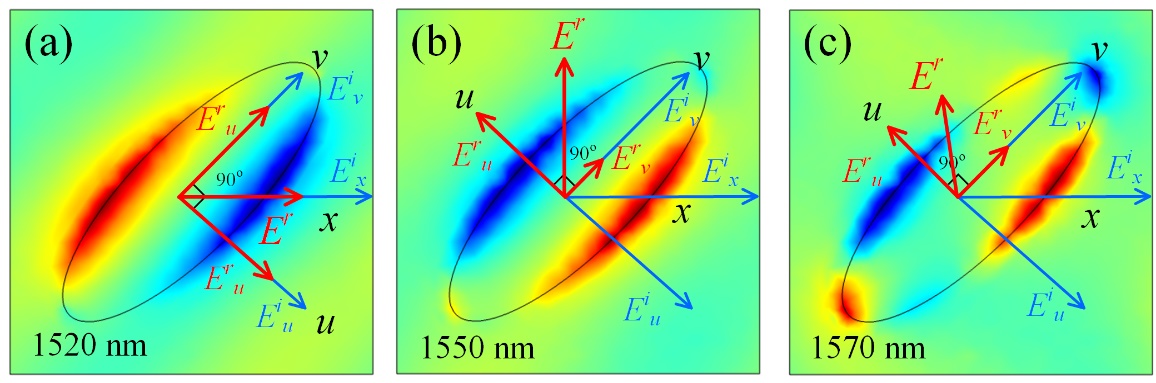


Fig. 6. Distribution of the z-component of the electric field (*E_z_*) on the graphene layer for normal x-polarization incidence at (a) 1520 nm, (b) 1550 nm, and (c) 1570 nm. In Fig. 5 and 6, a and b in *E^a^_b_* denote the incident (*i*) or reflection (*r*) and the polarization status.

Besides, for an x-polarization incidence, z-component electric field distributions on the graphene surface, at 1520, 1550, and 1570 (*nm*), are extracted and shown in Fig. 6, where the localized surface plasmon (LSP) is efficiently excited around the ellipse pattern forming electric dipoles [50, 51]. For 1520 nm, the LSP is along the horizontal direction parallel to the incident, and no conversion occurs. While for 1550 nm, the induced dipole is mainly along the *u-*axis and has a response to the *u*-component of the incident dramatically. Moreover, for the situation of 1570 nm, electric dipoles are efficiently generated in both the *u* and *v* axis and have different responses to both components. Furtherly, with recoupling, the reflection's amplitude and polarization status can be modulated, realizing a converter.

1. **The geometric dependences of the modulator**


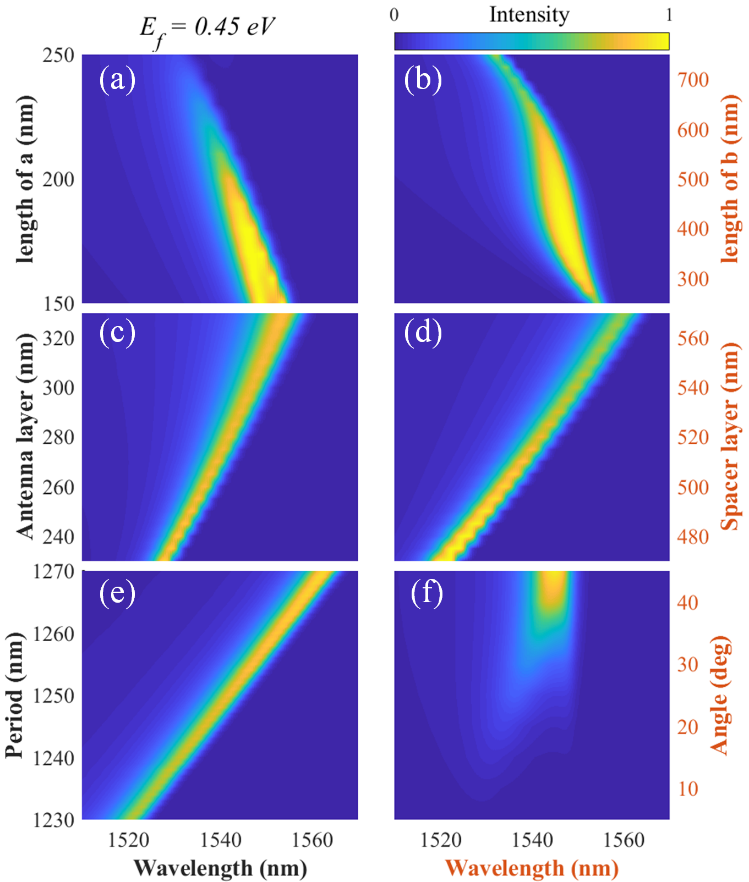


Fig. 7. The calculated dependence of PCR on the design parameters: (a) semi-minor axis of ellipse *a*; (b) semi-major axis of ellipse *b*; (c) the thickness of patterned antenna layer *h_1_*; (d) the thickness of spacer layer *h_2_*; (e) the periodicity of ellipse *P*; (f) the angle of elliptical patch *θ*. For each result, the calculations were performed starting from a set of design parameters, i.e., *a*=200 *nm*, *b*=600 *nm*, *h_1_*=280 *nm*, *h_2_*=520 *nm*, *P*=1250 *nm*, then only one parameter was changed. Here the angle rotated counterclockwise from the x-axis is defined as the elliptical angle *θ*.

Setting *E_f_* to be 0.45 *eV* and 0.3 *eV*, the corresponding PCR and graphene absorption spectra with different geometric parameters are calculated and shown in Fig. 7 and 8, respectively. The comparison analysis reveals the following conclusions:

1. Similar geometric dependencies are found. Therefore, a switchable modulator, i.e., absorber or polarization converter, can be achieved at a certain wavelength or over a broadband spectrum.
2. Antenna parameters *a* and *b* play essential roles in determining the operation wavelength and spectrum of the modulator. A broadband light modulator can be achieved using *a* of about 150 *nm* and *b* of about 420 *nm*.
3. If we tailor the parameters, including *h_1_*, *h_2_*, and *P*, the operation wavelength and spectrum of the modulator can be effectively tuned, mainly due to the size effect [42].
4. The comparison of Fig. 7(f) and 8(f) reveals that the change of the ellipse’s angle *θ* can shift the absorption spectrum, while the PCR spectrum only obtains a wideband peak at 45 degrees.


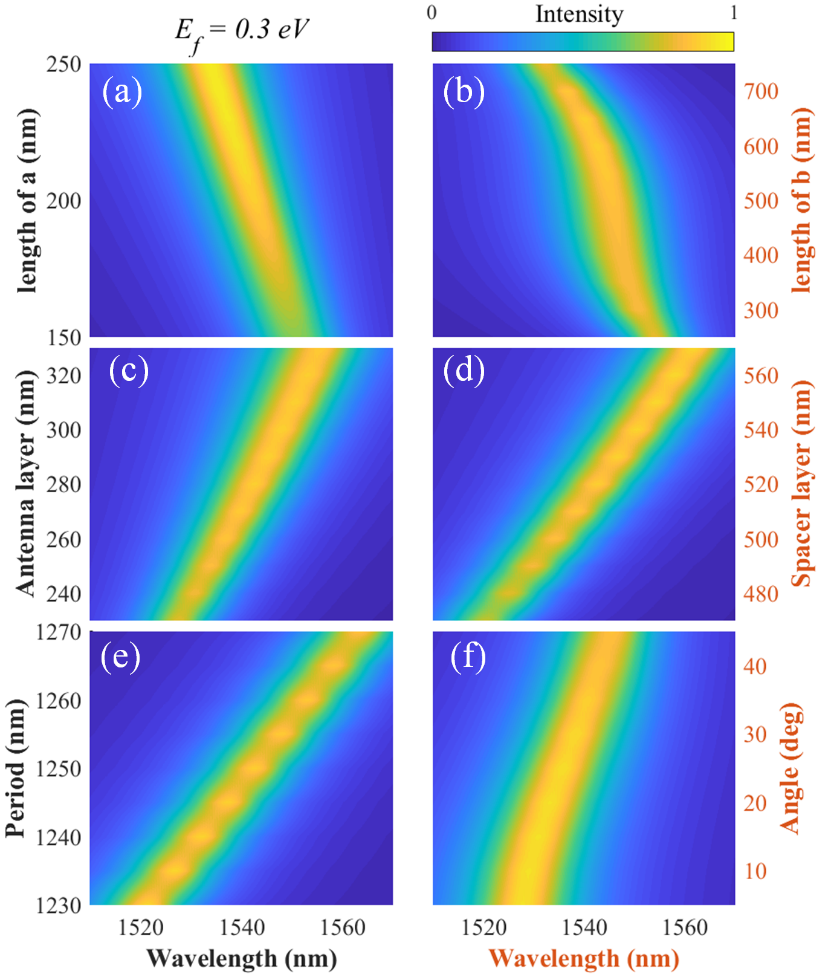


Fig. 8. The dependence of graphene absorption on the geometric parameters: short (a) and long (b) axis of the ellipse pattern, the thickness of antenna (c), silica layer (d), the period (e), and the rotation angle (f). Due to the symmetry, only calculate the angle in 0-45 degrees range.

1. **Electrical tunability of the modulator over a broad spectrum**

To further illustrate the electrical tunability of our device, a broadband modulator at 1550 nm domain is achieved with geometric parameters such as *a*= 180 *nm*, *b*=435 *nm*, *h_1_*=325 *nm*, *h_2_*=490 *nm*_,_ *P*=1250 *nm*, and *θ*=45 degrees. Since the tunability is realized through adjusting *E_f_*, Fig. 9(a) shows the graphene absorption at different Fermi energies. Meanwhile, the responding reflection coefficients, i.e., *R_xx_* and *R_yx_*, are illustrated in Fig. 9(b)-(g). Readily observation shows the declined graphene absorption and gradually raised *R_yx_*. The comparison of the situation when *E_f_*=0.41 *eV* to that when *E_f_*=0.45 *eV* reveals that the monolayer graphene performs as a lossless material (Imag(*ε_gra_*)=0), while a blueshift occurs in the reflection spectrum. The corresponding PCR spectra also experience a blueshift, shown in Fig. 9(h). These shifts are due to the Fermi-energy-dependent dispersion of graphene (see Fig. 3(a)), indicating a tunable operation spectrum of the modulator when working as a polarization converter. Moreover, Fig. 9(h) shows a PCR of over 90%, indicating an efficient polarization conversion.


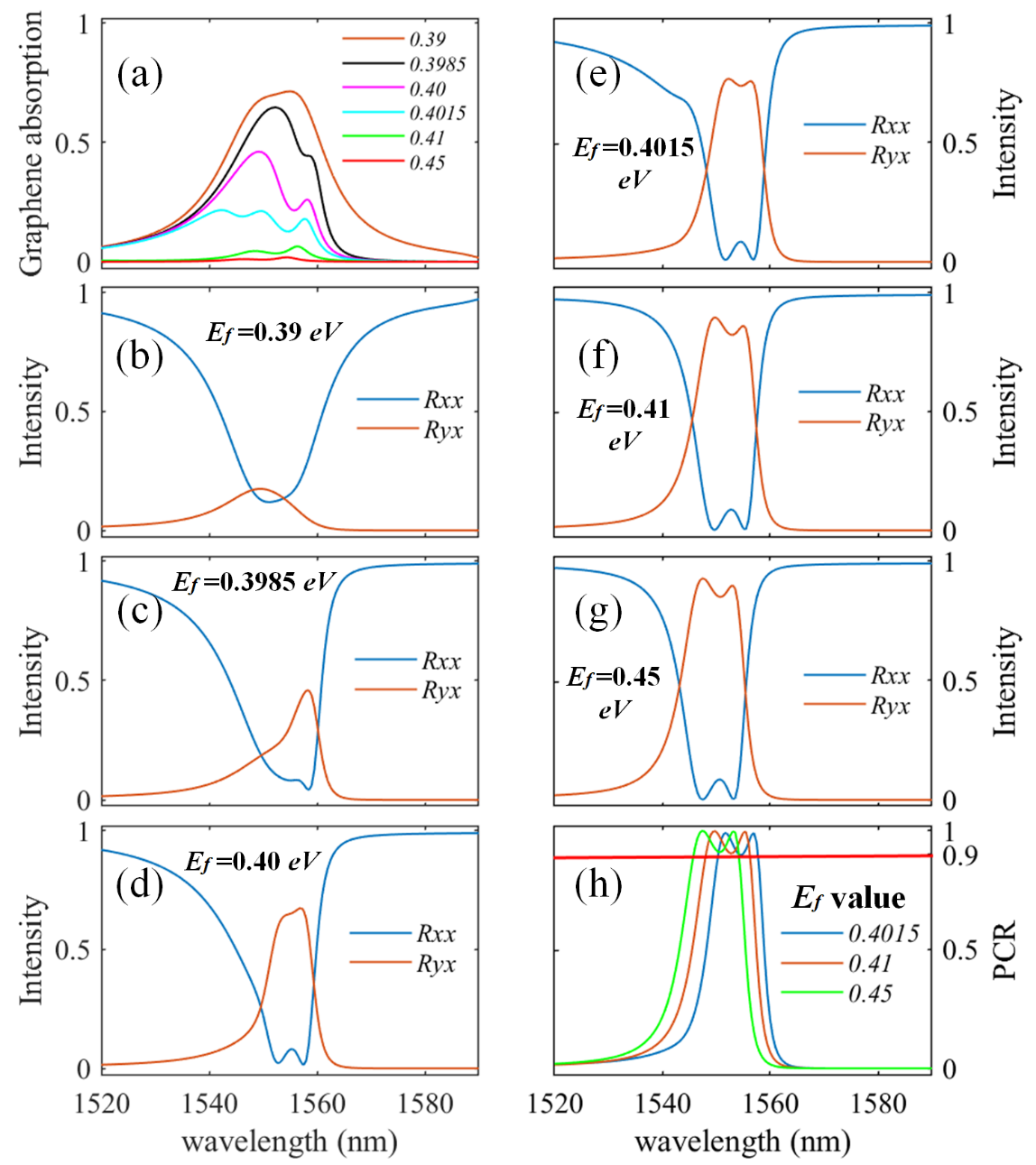


Fig. 9. (a) Numerically calculated absorption of the monolayer graphene sheet at different Fermi energy. *R_xx_* and *R_yx_* for the anisotropic meta-structure at Fermi energy of 0.39 *eV* (b), 0.3985 *eV* (c), 0.40 *eV* (d). The polarization conversion ratio at 0.4015 *eV*, 0.41 *eV*, and 0.45 *eV* are shown in (e), (f), and (g), respectively, and the corresponding PCRs are illustrated in (h).

When operating our device at 1550 *nm*, the reflection coefficients are calculated by tuning *E_f_* from 0.39 *eV* to 0.41 *eV*. As shown in Fig. 10(a), the *R_yx_* curve demonstrates a dramatical increase along with a sharp decline in the absorption curve, while *R_xx_* keeps relatively stable. These changes in respective curves reveal the monolayer graphene sheet changing from an absorptive material to a lossless dielectric. It is noted that these dramatic changes in curves happen in a relatively narrow *E_f_* range from 0.398 *eV* to 0.407 *eV*. With the dependence of *E_f_* on bias-gate-voltage described as *E_f_ = ℏV_F_*[*π|a_o_*(*V_g_-V_Dirac_*)|]*^1/2^*, fine voltage management is required to realize the tunability. Here *V_Dirac_* and *V_F_* represent the voltage offset and Fermi velocity of Dirac fermions in the graphene [25]. Corresponding PCR and the PD are shown in Fig. 10(b), depicting a high modulation depth between 52%-100% and an adjustable phase range from -71 degrees to 61 degrees.


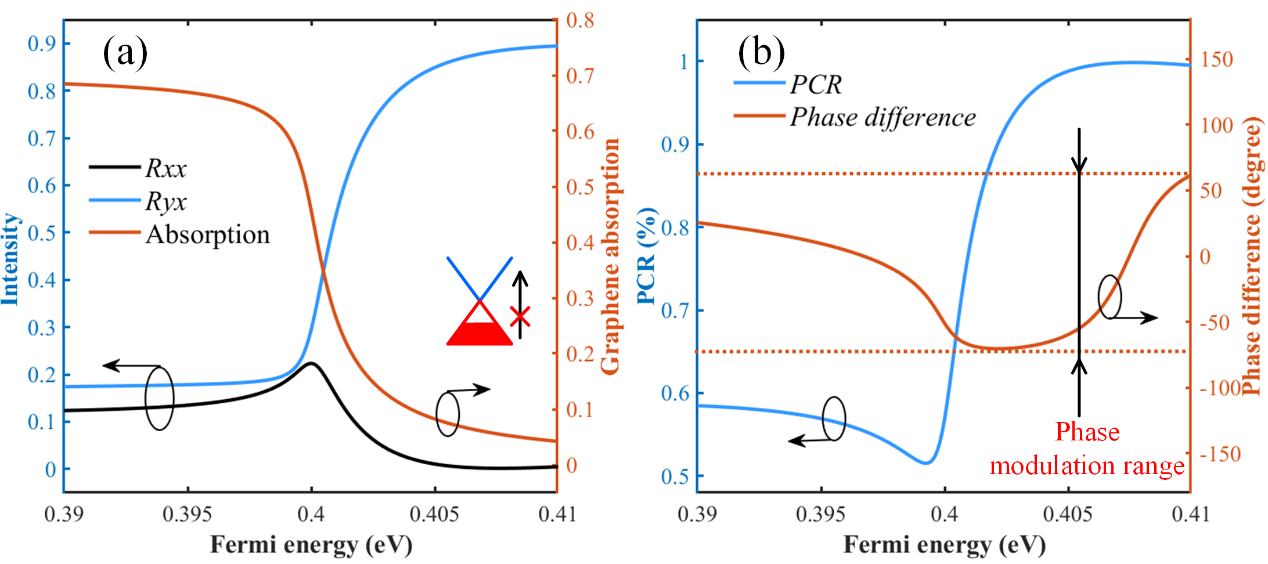


Fig. 10. (a) *R_xx_*, *R_yx_*, and graphene absorption (red line) at 1550 nm with tuning Fermi energy from 0.39 to 0.41 with a step of 5×10^-4^ *eV*. (b) corresponding PCR and phase difference (PD) between *R_xx_* and *R_yx_*.

The proposed modulator can be conveniently fabricated using well-established semiconductor techniques and procedures. The thick silver layer and silica spacer can be deposited on a substrate using the magnetron sputtering technique. Then the monolayer graphene sheet grown by chemical vapor deposition (CVD) is transferred onto the silica layer. 1D polymethy1-methacrylate material can fabricate the antenna block, which is further patterned using electron beam lithography and oxygen plasma etching [39, 52, 53]. In order to adjust the Fermi energy, Au electrodes can be formed on the top of the graphene sheet while using the bottom metal layer as the back gate [54, 55]. Thus, by changing the bias voltage applied between the monolayer graphene sheet and silver substrate, the Fermi energy can be actively tuned, realizing the device’s electrical switchability and tunability. It is worthwhile to note that the proposed graphene device needs a stable voltage turning with high precision and using E-beam lithography may impose limitations on the fabrication scalability.

1. **Conclusion**

This work studies a graphene metasurface-based infrared light modulator of electrical switchability and tunability. It is realized by tuning graphene’s Fermin energy and adjusting its optical dielectric coefficients, i.e., the imaginary and real parts. The proposed modulator can switch its function between an absorber and polarization converter by changing the monolayer graphene sheet from a lossy state (when Imag(*ε_gra_*)=0) to a lossless state (when Imag(*ε_gra_*)≠0). Furthermore, tuning *E_f_* can also tailor the operation spectrum of the polarization converter by leveraging the Fermi-energy-dependent dispersion of graphene, i.e., Real(*ε_gra_*). Our design may lead to an active light modulator of remarkable versatility and provides a new strategy for designing novel optoelectronic devices.

**Funding**

This work was supported by the Research Grants Council (RGC) of Hong Kong SAR (ECS24211020, GRF14203821, GRF14216222), the Innovation and Technology Fund (ITF) of Hong Kong SAR (ITS/240/21).

**Declaration of Competing Interest**

The authors declare no conflicts of interest.

**Reference**

1. Chen, D., et al., *The novel graphene metasurfaces based on split-ring resonators for tunable polarization switching and beam steering at terahertz frequencies.* Carbon, 2019. **154**: p. 350-356.

2. Zhang, Y., Y. Feng, and J. Zhao, *Graphene-enabled tunable multifunctional metamaterial for dynamical polarization manipulation of broadband terahertz wave.* Carbon, 2020. **163**: p. 244-252.

3. Zeng, F., et al., *Tunable mid-infrared dual-band and broadband cross-polarization converters based on U-shaped graphene metamaterials.* Optics Express, 2019. **27**(23): p. 33826-33839.

4. Ghosh, S.K., S. Das, and S. Bhattacharyya, *Terahertz Wave Conversion from Linear to Circular Polarization by Graphene Metasurface Featuring Ultrawideband Tunability.* Journal of Lightwave Technology, 2022.

5. Huang, C.p., et al., *Break through the limitation of Malus' law with plasmonic polarizers.* Advanced Optical Materials, 2014. **2**(8): p. 723-728.

6. Ali, T., et al., *On‐Demand Polarization Controllable Liquid Crystal Laser.* Advanced Materials Technologies, 2022: p. 2200674.

7. Rahnama, A., et al., *In‐Fiber Switchable Polarization Filter Based on Liquid Crystal Filled Hollow‐Filament Bragg Gratings.* Advanced Optical Materials, 2021. **9**(19): p. 2100054.

8. Kan, Y., et al., *Metasurface‐enabled generation of circularly polarized single photons.* Advanced Materials, 2020. **32**(16): p. 1907832.

9. Ding, F., et al., *Metasurface-enabled broadband beam splitters integrated with quarter-wave plate functionality.* Nanoscale, 2020. **12**(26): p. 14106-14111.

10. Fahad, A.K., et al., *Triple-wide-band ultra-thin metasheet for transmission polarization conversion.* Scientific Reports, 2020. **10**(1): p. 1-12.

11. Zou, M., M. Su, and H. Yu, *Ultra-broadband and wide-angle terahertz polarization converter based on symmetrical anchor-shaped metamaterial.* Optical Materials, 2020. **107**: p. 110062.

12. Huang, X., et al., *Simultaneous realization of polarization conversion for reflected and transmitted waves with bi-functional metasurface.* Scientific reports, 2022. **12**(1): p. 1-10.

13. Momeni, A., K. Rouhi, and R. Fleury, *Switchable and simultaneous spatiotemporal analog computing with computational graphene-based multilayers.* Carbon, 2022. **186**: p. 599-611.

14. Deng, Y., et al., *Multifunctional graphene metadevice using dual metamaterial induced transparency in terahertz regime.* Carbon, 2022.

15. Heidari, S. and N. Nozhat, *Graphene-based triple-band switch with circuit model method in far-infrared region.* Optical and Quantum Electronics, 2022. **54**(3): p. 13.

16. Gong, Y., et al., *Terahertz binary coder based on graphene metasurface.* Carbon, 2021. **184**: p. 167-176.

17. Zeng, C., et al., *Graphene-empowered dynamic metasurfaces and metadevices.* Opto-Electronic Advances, 2022. **5**(4): p. 200098-1-200098-47.

18. Nong, J., et al., *Wideband tunable perfect absorption of graphene plasmons via attenuated total reflection in Otto prism configuration.* Nanophotonics, 2020. **9**(3): p. 645-655.

19. Zhao, W., et al., *Observation of hydrodynamic plasmons and energy waves in graphene.* Nature, 2023. **614**(7949): p. 688-693.

20. Fan, K., R.D. Averitt, and W.J. Padilla, *Active and tunable nanophotonic metamaterials.* Nanophotonics, 2022. **11**(17): p. 3769-3803.

21. Li, Q., et al., *Gate-tuned graphene meta-devices for dynamically controlling terahertz wavefronts.* Nanophotonics, 2022. **11**(9): p. 2085-2096.

22. Feng, M., et al., *Active metal–graphene hybrid terahertz surface plasmon polaritons.* Nanophotonics, 2022. **11**(14): p. 3331-3338.

23. Ansell, D., et al., *Hybrid graphene plasmonic waveguide modulators.* Nature Communications, 2015. **6**: p. 6.

24. Bao, Q. and K.P. Loh, *Graphene photonics, plasmonics, and broadband optoelectronic devices.* ACS nano, 2012. **6**(5): p. 3677-3694.

25. Liu, M., et al., *A graphene-based broadband optical modulator.* Nature, 2011. **474**(7349): p. 64-67.

26. Dai, Z., et al., *Quantum-Well Bound States in Graphene Heterostructure Interfaces.* Physical Review Letters, 2021. **127**(8): p. 086805.

27. Lu, H., et al., *Tunable high-efficiency light absorption of monolayer graphene via Tamm plasmon polaritons.* Optics Letters, 2016. **41**(20): p. 4743-4746.

28. Yan, Z., et al., *Ultra-broadband and completely modulated absorption enhancement of monolayer graphene in a near-infrared region.* Optics Express, 2022. **30**(19): p. 34787-34796.

29. Chen, Z., et al., *Graphene controlled Brewster angle device for ultra broadband terahertz modulation.* Nature communications, 2018. **9**(1): p. 1-7.

30. Grigorenko, A.N., M. Polini, and K. Novoselov, *Graphene plasmonics.* Nature photonics, 2012. **6**(11): p. 749-758.

31. Kumar, R., et al., *Heteroatom doped graphene engineering for energy storage and conversion.* Materials Today, 2020. **39**: p. 47-65.

32. Chen, J., et al., *Broadband, wide-incident-angle, and polarization-insensitive high-efficiency absorption of monolayer graphene with nearly 100% modulation depth at communication wavelength.* Results in Physics, 2022. **40**: p. 105833.

33. Mou, N., et al., *Hybridization-induced broadband terahertz wave absorption with graphene metasurfaces.* Optics Express, 2018. **26**(9): p. 11728-11736.

34. Ye, L., et al., *Composite graphene-metal microstructures for enhanced multiband absorption covering the entire terahertz range.* Carbon, 2019. **148**: p. 317-325.

35. Ye, L., et al., *Frequency-reconfigurable wide-angle terahertz absorbers using single-and double-layer decussate graphene ribbon arrays.* Nanomaterials, 2018. **8**(10): p. 834.

36. Nong, J., et al., *Combined Visible Plasmons of Ag Nanoparticles and Infrared Plasmons of Graphene Nanoribbons for High‐Performance Surface‐Enhanced Raman and Infrared Spectroscopies.* Small, 2021. **17**(1): p. 2004640.

37. Gao, F., et al., *Broadband wave absorption in single-layered and nonstructured graphene based on far-field interaction effect.* Optics Express, 2017. **25**(9): p. 9579-9586.

38. Chen, X., et al., *All-dielectric metasurfaces with high Q-factor Fano resonances enabling multi-scenario sensing.* Nanophotonics, 2022. **11**(20): p. 4537-4549.

39. Guo, C.C., et al., *Experimental demonstration of total absorption over 99% in the near infrared for monolayer‐graphene‐based subwavelength structures.* Advanced Optical Materials, 2016. **4**(12): p. 1955-1960.

40. Qing, Y.M., H.F. Ma, and T.J. Cui, *Flexible control of light trapping and localization in a hybrid Tamm plasmonic system.* Optics Letters, 2019. **44**(13): p. 3302-3305.

41. Qing, Y.M., et al., *Near-infrared absorption-induced switching effect via guided mode resonances in a graphene-based metamaterial.* Optics Express, 2019. **27**(4): p. 5253-5263.

42. Chen, J., et al., *Electrically modulating and switching infrared absorption of monolayer graphene in metamaterials.* Carbon, 2020. **162**: p. 187-194.

43. Nong, J., et al., *Active Modulation of Graphene Near‐Infrared Electroabsorption Employing Borophene Plasmons in a Wide Waveband.* Advanced Optical Materials, 2022. **10**(6): p. 2102131.

44. Xu, C., et al., *Characteristics of electro-refractive modulating based on Graphene-Oxide-Silicon waveguide.* Optics express, 2012. **20**(20): p. 22398-22405.

45. Thongrattanasiri, S., F.H.L. Koppens, and F.J. García de Abajo, *Complete Optical Absorption in Periodically Patterned Graphene.* Physical Review Letters, 2012. **108**(4): p. 047401.

46. Lu, H., et al., *Graphene-supported manipulation of surface plasmon polaritons in metallic nanowaveguides.* Photonics Research, 2017. **5**(3): p. 162-167.

47. Tang, H., et al., *Photo-modulated optical and electrical properties of graphene.* Nanophotonics, 2022. **11**(5): p. 917-940.

48. Wang, F., et al., *Gate-variable optical transitions in graphene.* science, 2008. **320**(5873): p. 206-209.

49. Ren, L., et al., *Terahertz and infrared spectroscopy of gated large-area graphene.* Nano Letters, 2012. **12**(7): p. 3711-3715.

50. Wang, Y., et al., *Circular Dichroism Enhancement and Biosensing Application of Composite Dielectric Chiral Nanostructures.* The Journal of Physical Chemistry C, 2021. **125**(45): p. 25243-25252.

51. Chen, J., et al., *Dielectric waveguide-enhanced localized surface plasmon resonance refractive index sensing.* Optical Materials Express, 2018. **8**(2): p. 342-347.

52. Gao, Z., et al., *Large-area epitaxial growth of curvature-stabilized ABC trilayer graphene.* Nature communications, 2020. **11**(1): p. 1-10.

53. Yeung, K.K., et al., *Utilizing Gradient Porous Graphene Substrate as the Solid-Contact Layer To Enhance Wearable Electrochemical Sweat Sensor Sensitivity.* Nano Letters, 2022. **22**(16): p. 6647-6654.

54. Kumar, J., et al., *Carbon Vacancy Assisted Contact Resistance Engineering in Graphene FETs.* IEEE Transactions on Electron Devices, 2022. **69**(4): p. 2066-2073.

55. Li, J., et al., *Aptasensors Based on Graphene Field-Effect Transistors for Arsenite Detection.* ACS Applied Nano Materials, 2022. **5**(9): p. 12848-12854.
